# A Method of Human-Like Compliant Assembly Based on Variable Admittance Control for Space Maintenance

**DOI:** 10.34133/cbsystems.0046

**Published:** 2023-09-06

**Authors:** Xiaolei Cao, Xiao Huang, Yan Zhao, Zeyuan Sun, Hui Li, Zhihong Jiang, Marco Ceccarelli

**Affiliations:** ^1^ School of Mechatronical Engineering, Advanced Innovation Center for Intelligent Robots and Systems, Key Laboratory of Biomimetic Robots and Systems of Chinese Ministry of Education, Beijing Institute of Technology, Beijing 100081, China.; ^2^ China Northern Vehicle Research Institute, Beijing 100071, China.; ^3^ Department of Industrial Engineering, University of Rome Tor Vergata, Via del Politecnico 1, 00133 Roma, Italy.

## Abstract

On-orbit assembly has become a crucial aspect of space operations, where the manipulator frequently and directly interacts with objects in a complex assembly process. The traditional manipulator control has limitations in adapting to diverse assembly tasks and is vulnerable to vibration, leading to assembly failure. To address this issue, we propose a human-like variable admittance control method based on the variable damping characteristics of the human arm. By collecting the velocity and contact force of human arm operations in assembly, we analyze the damping change of human arm and establish the active compliance model based on S-type damping variation rule in assembly. Furthermore, 3 passive contact models are proposed between the end of the human arm and the environment: one-sided bevel contact, both sides bevel contact, and pin–hole contact. On the basis of these active and passive models, a typical space assembly task for a robot is designed, and a human-like variable admittance controller is established and simulated. Finally, we build a ground verification platform and complete different assembly tasks, thereby successfully verifying the safety, robustness, and adaptability of the human-like variable admittance control method.

## Introduction

Robots are increasingly being used for maintenance and repair in space due to their greater adaptability to the harsh space environment compared to human astronauts [1–3]. This trend is critical for the development of space technology, as it can help mitigate health risks for humans in space stations and address the challenges of repairing spacecraft in space [4–7]. Robotic assembly is a vital field of research that has seen important progress in recent years. Compliance control has emerged as the primary method for enabling robots to perform complex assembly tasks. However, compliance control places high demands on the manipulator’s contact performance, making it challenging to achieve the required levels of precision and adaptability [8–11].

In response to these challenges, researchers have proposed various compliance control methods, including damping control, stiffness control, force/position hybrid control, and fuzzy adaptive control algorithms [12–18]. These algorithms aim to improve the adaptability and efficiency of robots when performing assembly tasks, particularly in unknown environments. For example, a forgetting factor-based human-like compliance control algorithm that enabled autonomous and stable assembly of a 6-pin and 6-hole load plate disk outside the space station cabin was introduced [19–21]. Meanwhile, Ren et al. [22] proposed an optimized adaptive control algorithm that achieved mixed position and force tracking even in uncertain closed chain motion. Furthermore, Wu et al. [23] presented a hybrid control method of force position based on servo speed loop for high-precision shaft hole assembly.

Other researchers have explored the relationship between stiffness and arm motion during human arm movements. For instance, Mo et al. [24] introduced a human-like contact compliance control algorithm based on the changing characteristics of human wrist stiffness, thus allowing the robot to perform dexterous operations. Burdet et al. [25] suggested that increasing stiffness could reduce interference in arm motion. Yang et al. [26] presented a human-like adaptive robot control method that enabled human-like variable impedance control of robots in unknown environments. Moreover, Kang et al. [27] proposed a variable admittance control method to achieve intuitive human–robot interactions that consider human intentions by incorporating direct and indirect human intent. Dimeas and Aspragathos [28] proposed a method for variable admittance control in human–robot cooperation tasks, which combines a human-like decision-making process and an adaptation algorithm. Despite these developments, robotic assembly based on compliance control still faces challenges, such as inadequate adaptability to complex assembly tasks. Addressing these limitations is imperative for the efficient control of robotic assembly. In this paper, we propose a new classification of compliance control methods based on their strengths and limitations.

This study introduces a compliant variable parameter admittance control approach that imitates the variable damping characteristics of the human arm to enhance the adaptability of a manipulator in diverse assembly tasks. The method aims to provide the manipulator with dynamic features comparable to those of the human arm, thereby allowing it to execute assembly tasks with similar stability and efficiency. Our study’s primary objective is to propose a human-like compliant variable parameter admittance control technique that facilitates the manipulator’s adaptability in diverse assembly tasks. The contributions of the present study are as follows:

1. This study collects contact force and velocity data of a human arm during assembly tasks to acquire its variable damping characteristics. Subsequent analysis reveals that the contact process between the human arm and the environment comprises 3 subprocesses: proximity, contact, and compaction. Using these subprocesses as a foundation, we establish contact models for active and passive compliance in human arm assembly.
2. This study proposes a compliant variable parameter admittance control approach that mimics the variable damping characteristics of the human arm. The controller utilizes an admittance control framework, incorporating the active variable damping characteristics of the human arm and integrating passive contact models of different states. In addition, the controller incorporates dynamic planning of a balance position based on a forgetting factor, which reduces contact force during assembly tasks.

The remainder of this paper is structured as follows. Dynamic Modeling of Human Assembly section focuses on the collection and analysis of the dynamic characteristics of the human arm during assembly tasks and establishes a dynamic guidance model. Simulation Experiments section presents the simulation results for the manipulator satellite assembly, thus demonstrating the effectiveness of the proposed method. In Experiment Using a Ground Verification Platform section, we present the ground verification platform and the successful completion of various assembly tasks. Finally, Discussion section summarizes the study and outlines future research directions.

## Dynamic Modeling of Human Assembly

In this section, we aim to construct an experimental data acquisition platform to collect contact force and velocity data during assembly tasks performed by a human arm, with the objective of obtaining the arm’s variable damping characteristics. On the basis of subsequent analysis of the acquired data, we abstract the human arm as a viscoelastic system and establish a dynamic active compliance and passive contact model for human assembly.

### Acquisition and analysis of human arm assembly data

**Fig. 1.**
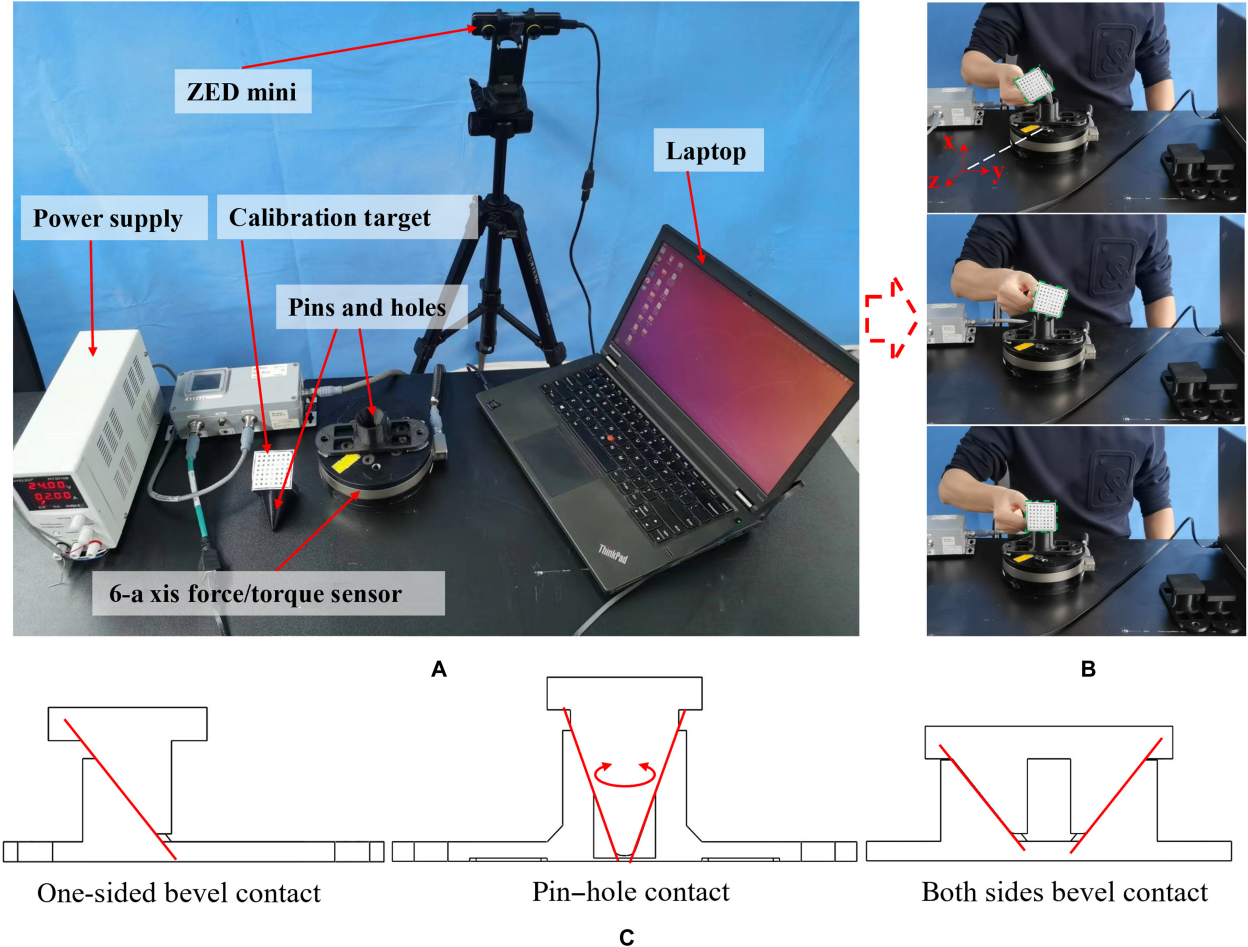
(A) Experimental setup for measuring human arm force and velocity, (B) acquisition process of damping characteristics experiment, and (C) 3 types of contact forms.

Assembly tasks frequently involve contact between a manipulator and an object being assembled. To prevent excessive contact force from damaging the object, damping is necessary to dissipate energy and restrain vibration. Objects with larger damping consume energy more rapidly under external forces. The human arm’s musculoskeletal system can flexibly adjust damping to perform various tasks safely and stably. To collect parameters such as contact force and velocity, we established a dynamic data acquisition platform to capture human arm motion (Fig. 1). The system’s main components include a motion capture subsystem and a contact force measurement subsystem. An ATI omega160 6D force sensor is used to collect contact force data between the human hand and assembly parts, while end velocity data of the human arm are obtained using a Stereolabs ZED mini motion capture system.

Space assembly involves various tasks, including satellite capture and docking, where component errors must be considered to improve task success rate. Through analysis of the assembly process, we identified 3 primary contact forms that play a critical role in assembly tasks: (a) pin–hole contact, (b) one-sided bevel contact; and (c) both sides bevel contact.

**Fig. 2.**
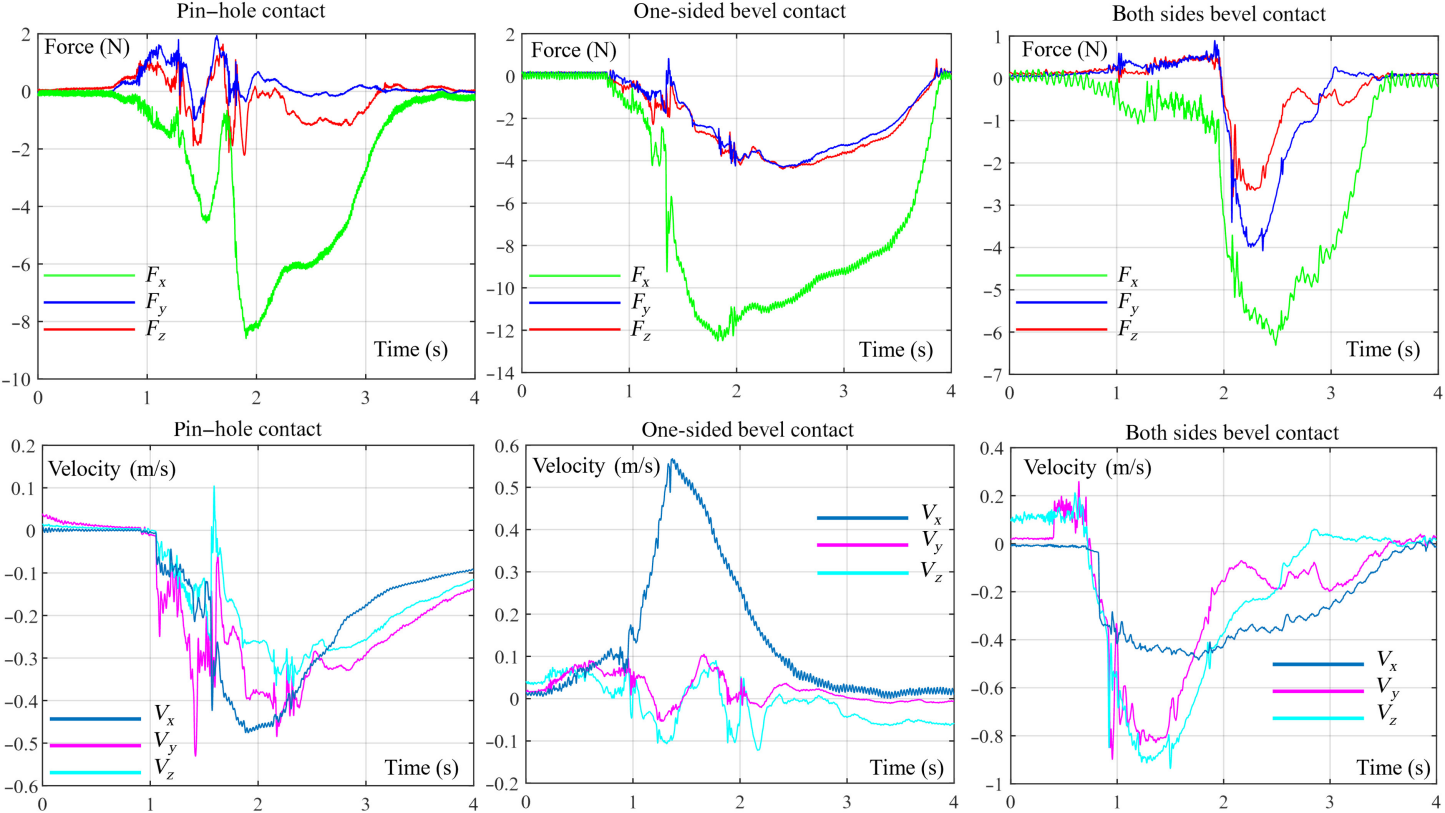
Curves of force and end velocity for assembly.

In our experiments, 3 different types of assemblies were utilized for assembly tasks, as depicted in Fig. 1C. The assembled parts were securely connected with an ATI Omega160 6-axis force/torque sensor to measure the contact force. In addition, the movement speed of the human arm’s end was measured using a Stereolabs ZED Mini vision system, as shown in Fig. 1A. To avoid any subjective influence on the end motion characteristics, we blindfolded the experimenter during the 3 distinct experiments, as shown in Fig. 1B. Data concerning the contact force and velocity of the human arm were obtained during the experiments (Fig. 2), thus providing the foundation for subsequent analysis and modeling of the human arm.

### Contact model of flexible assembly performed by human arm

**Fig. 3.**
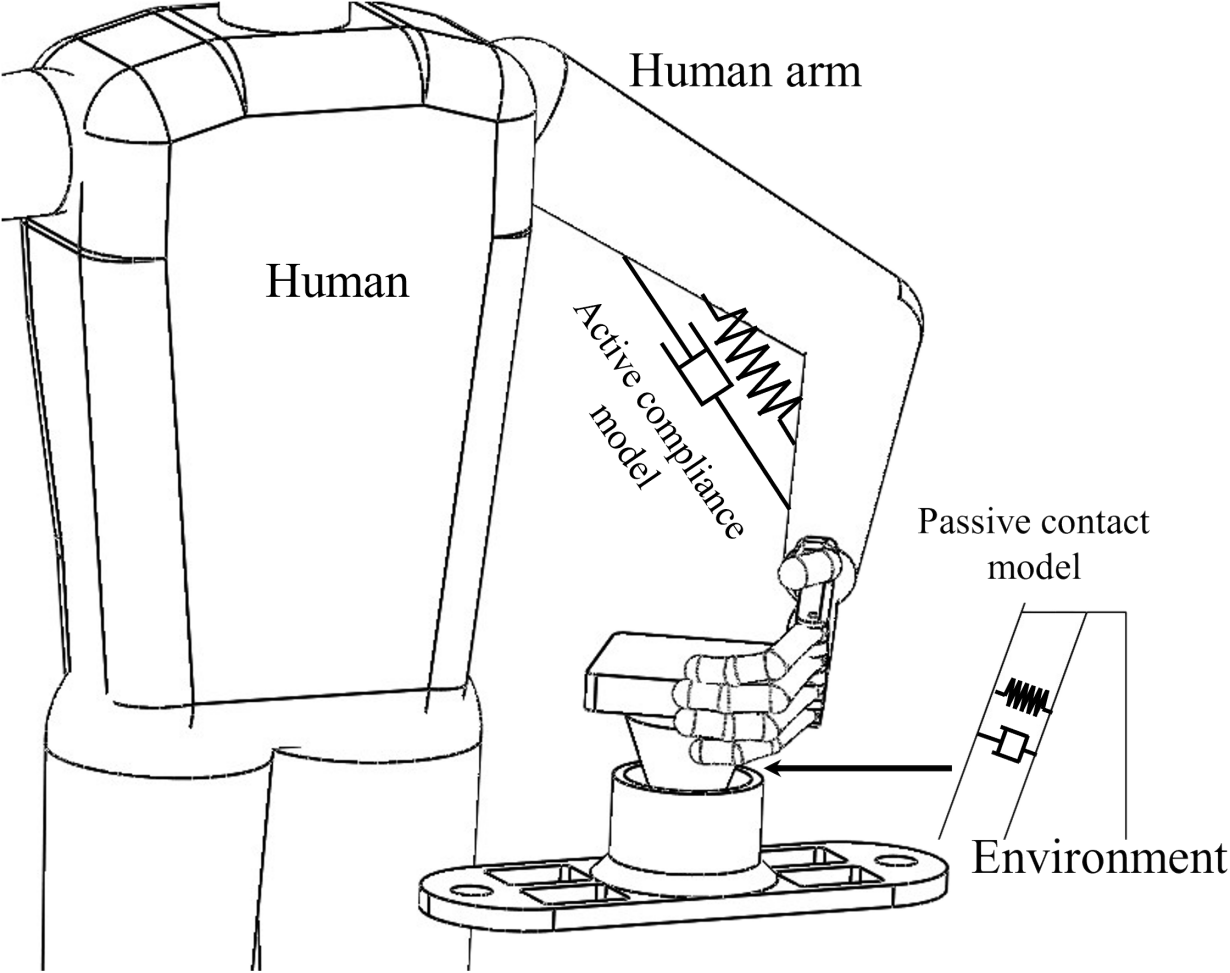
Compliant assembly model for a human arm.

The dynamic model for the assembly process involves 2 primary components: the active compliance model of the human arm and the passive contact model between the human arm’s end and the surrounding environment. This is illustrated in Fig. 3. In this study, the assembly processes of the pin–hole contact form, one-sided bevel contact form, and both sides bevel contact form were analyzed to derive the comprehensive model for compliant assembly using a human arm.

**Fig. 4.**
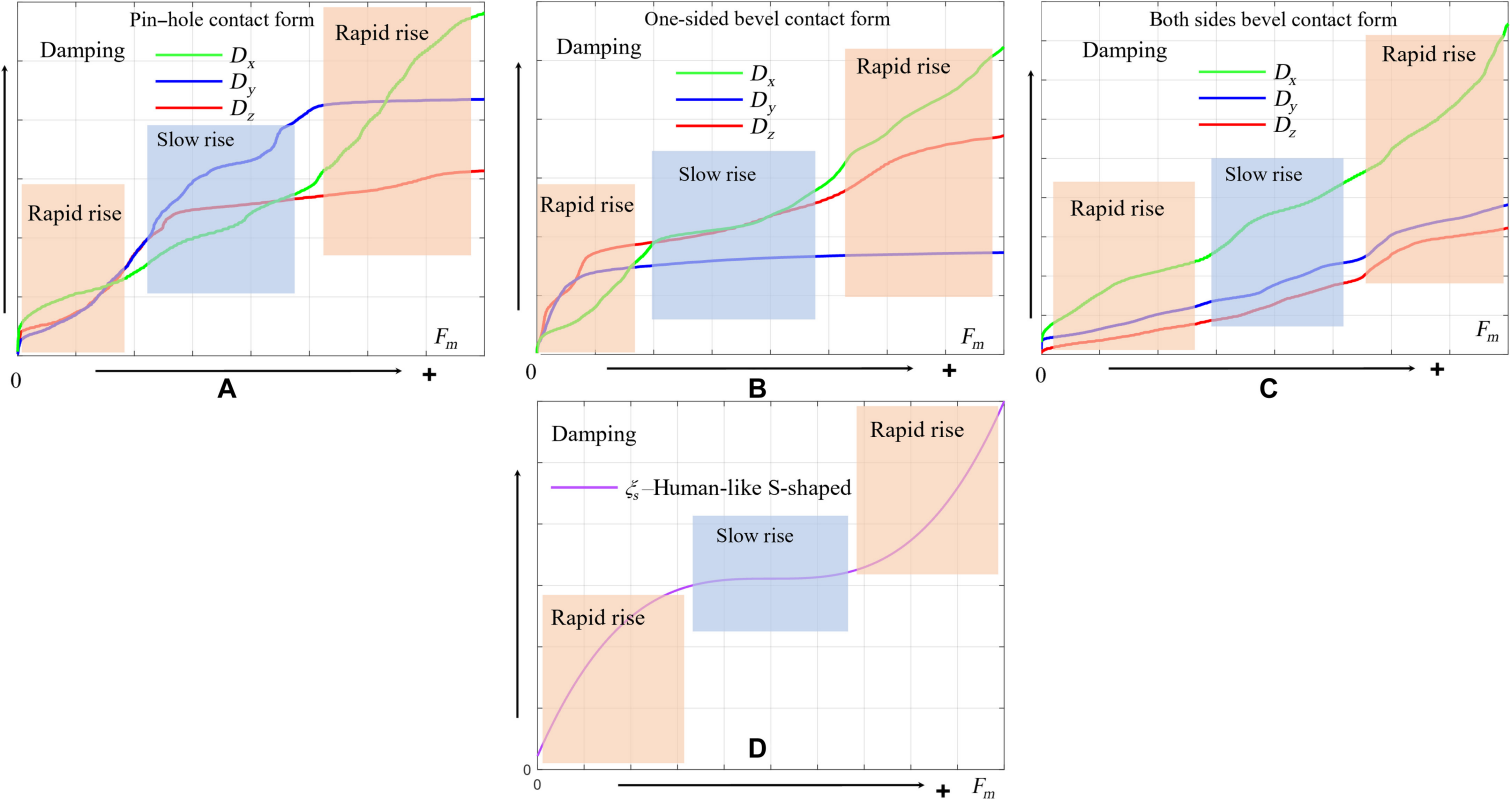
(A to C) Damping curves of the human arm in 3 different assembly tasks and (D) an S-shaped damping curve.

**Fig. 5.**
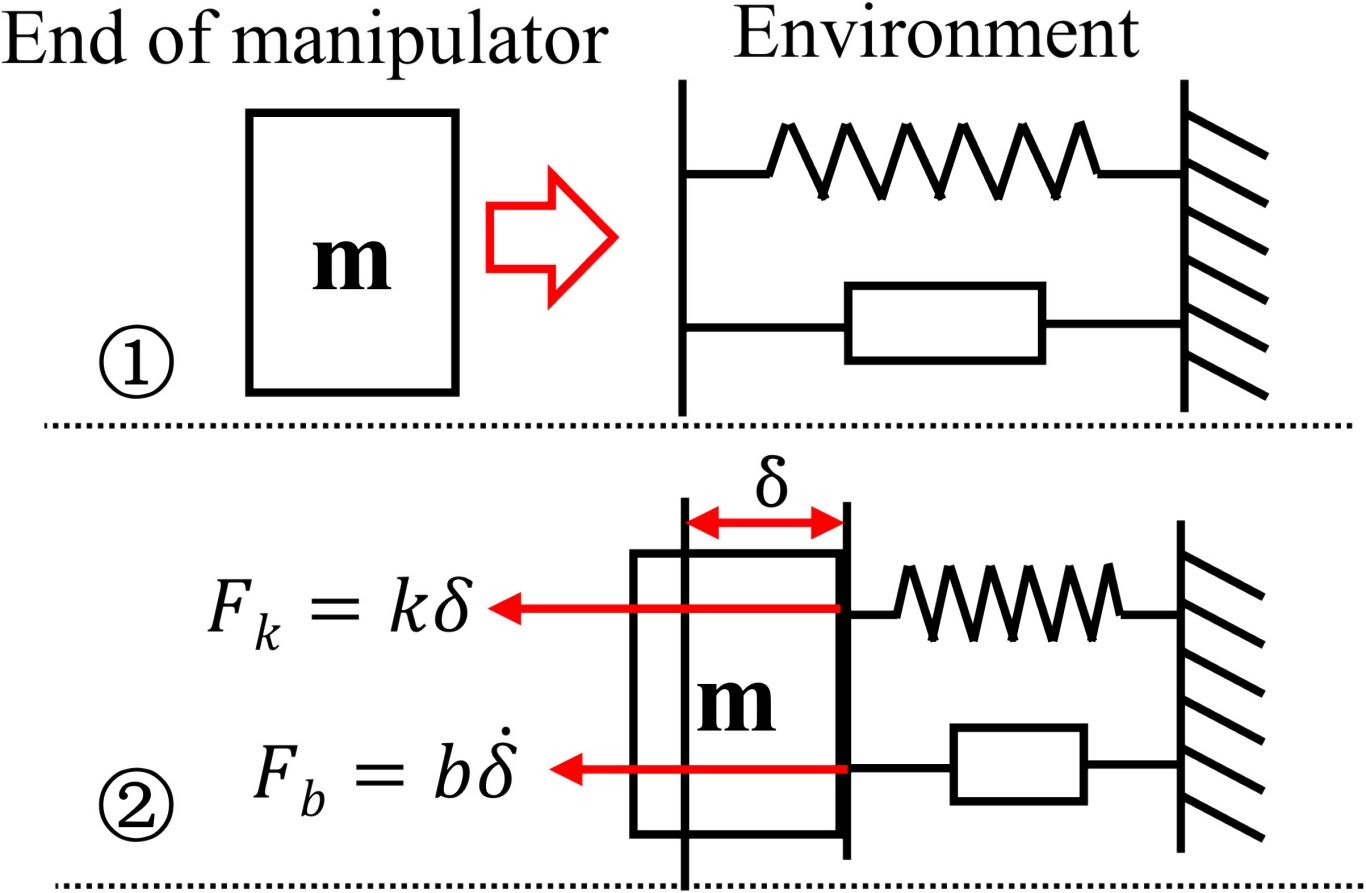
Contact process between the manipulator and environment.

1. Active compliance model. As shown in Fig. 3, according to the damping changing characteristic equation *F* = *CV*, the damping characteristic of the human arm can be calculated according to obtained data (Fig. 2), where *F* is the external force, *V* is the motion speed, and *C* is the damping. In the assembly tasks performed by the human arm, the collision caused by the initial error in the assembly process leads to a large internal force, and the damping of the human arm changes with the change in this internal contact force. The human arm will adjust the damping in the direction that will improve compliance. As shown in Fig. 4A to C, the damping of the human arm is positively correlated with the contact force, which is divided into 3 stages: rapid rise, slow rise, and rapid rise. During assembly tasks, people adjust their arm damping to maintain stability. They quickly adjust damping to an appropriate range when external forces are too small or too large. We fit the damping curve as an S-type curve and obtain the curve shown in Fig. 4D and expressed as Eq. 1. Here, *F_m_* is the maximum contact force, *D*_s_ is the active compliance damping, and *k_s_* is the fitting coefficient.
  $$D_{S}=k_{S}{\left(F-\frac{F_{m}}{2}\right)}^{3}+\frac{k_{s}{F_{m}}^{3}}{8}$$ (1)
2. Passive contact model. After conducting an analysis of the 3 assembly processes mentioned above, it was discovered that the contact process between the human arm and the surrounding environment consists of 3 distinct subprocesses: proximity, contact, and compaction. The modeling of these subprocesses will be conducive to the stable control of the whole process. The whole process is shown in Fig. 5.

The assembly parts exhibit high rigidity and low plasticity, making them similar to a high rigidity spring with minimal damping. To maintain the required positioning accuracy, we model the environment as a high-rigidity light spring system. The Hertz contact model [29] describes the relationship between contact force and indentation depth in pure elastic contact. This model is used to determine the contact force and deformation of the assembly parts during their interaction. By utilizing the Hertzian model to analyze contact mechanics, the deformation and stress distribution of assembled components can be obtained as follows:

$$F_{n}=k_{e}\delta^{n}=k_{\delta }\delta$$ (2)

where *k_e_* is the environmental stiffness, *F_n_* is the contact force, *δ* is the pressing depth, and *k_δ_* = *k_e_δ ^n−1^*. For metal materials, *F_n_* and *δ* are nonlinear.

**Fig. 6.**
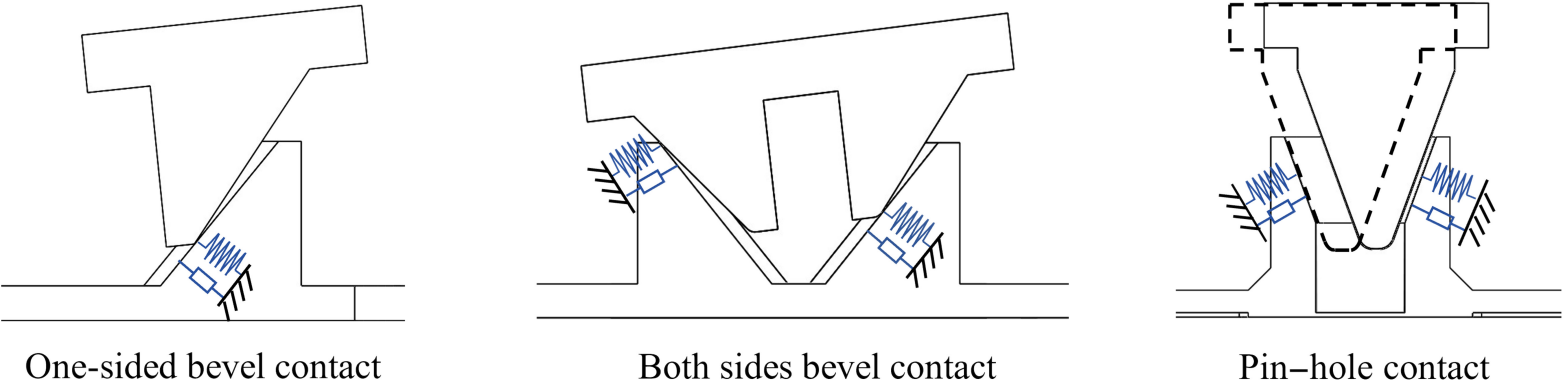
Analysis of passive contact modes.

On the basis of Eq. 2, the proposed model is capable of analyzing 3 distinct passive contact modes between the human arm and the environment. These modes are presented in Table 1. As shown in Fig. 6, in the case of the one-sided bevel contact form, the 2 inclined surfaces cannot be in complete contact due to errors during the docking process; this results in linear contact between them. Here, the 2 inclined surfaces can be treated as a one-sided spring system. In the case of the both sides bevel contact form, the 2 sides of the inclined surface are unable to achieve complete contact due to the docking process’ inherent errors, resulting in linear contact on both sides. This scenario can be regarded as a continuous both sides spring system. Regarding the pin–hole contact form, the presence of a gap between the pin–hole causes the pin to move back and forth within the hole. Therefore, the pin–hole docking mode can be considered as a 2-sided spring system. However, it should be noted that both sides do not act simultaneously in this case.

**Table 1.** Classification of contact models.

Contact form		Contact models
One side	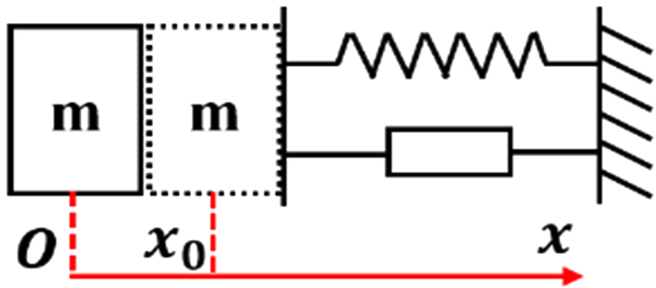	$F_{n}\left(x\right)=\left\{\begin{array}{cc} 0 & \left(x<x_{0}\right)\\ -k_{\delta }\left(x-x_{0}\right) & \left(x\geq x_{0}\right) \end{array}\right.$
Both sides	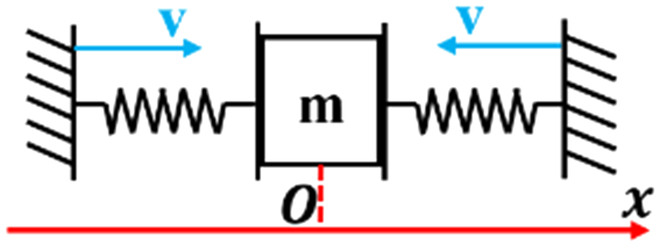	*F_n_*(*x*) = − *k_δ_x*
Pin–hole	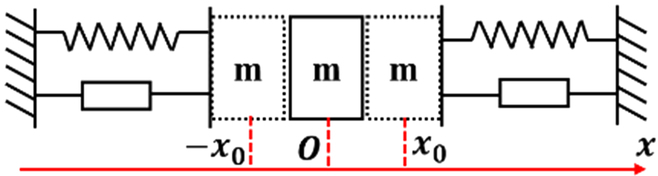	$F_{n}\left(x\right)=\left\{\begin{array}{cc} -k_{\delta }\left(x+x_{0}\right) & \left(x<-x_{0}\right)\\ 0 & \left(-x_{0}\leq x<x_{0}\right)\\ -k_{\delta }\left(x-x_{0}\right) & \left(x\geq x_{0}\right) \end{array}\right.$

On the basis of the principle that force and motion in the 6 directions at the end of the human arm are independent, it is possible to extend one-dimensional analysis to 6 dimensions.

On the basis of the analysis presented above, the human body can be understood as a complex system consisting of active compliance control in the human arm and passive compliance control between the end of the arm and the surrounding environment during assembly tasks. We thus propose a robot controller that utilizes active and passive compliance control principles to optimize performance in assembly tasks.

### Construction of human-like admittance controller

**Fig. 7.**
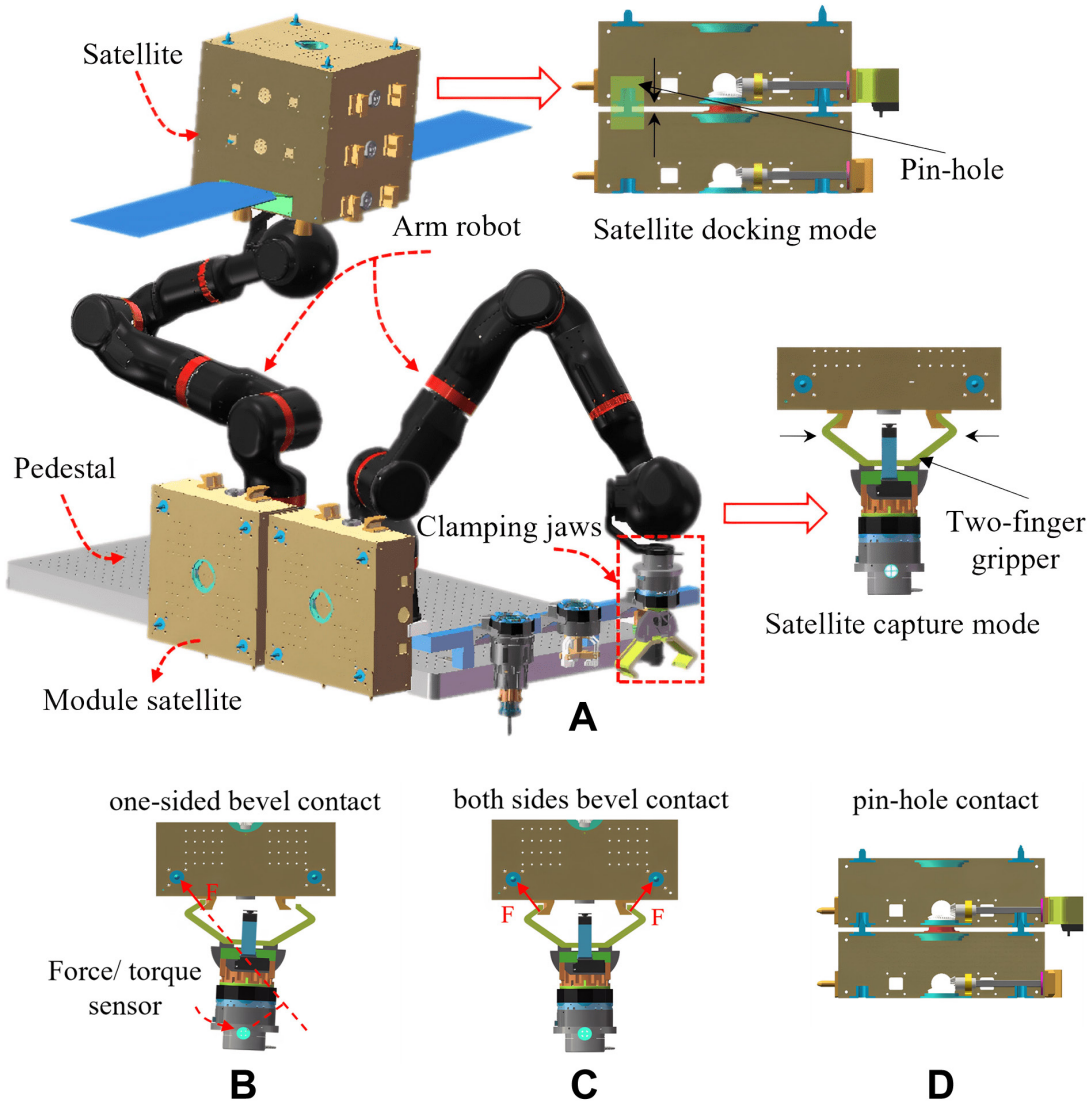
Satellite assembly task. (A) The process of satellite assembly. (B) One-finger contact between gripper and satellite module. (C) Two-finger contact between gripper and satellite module. (D) Pin–hole contact between satellite modules.

In this study, we present a simulation of a robot space assembly task involving a 7-degree-of-freedom manipulator that is responsible for grasping and docking a satellite module while in orbit. The manipulator uses a 2-finger gripper to initially grab the satellite module. This is followed by a docking process and finally the locking of the module through the pin–hole, as illustrated in Fig. 7A. The overall assembly task can be simplified into 3 distinct contact forms, which are shown in Fig. 7B to D.

The initial contact form between the 2-finger gripper and the satellite module is established through a single bevel contact. During the locking process, bilateral bevel contact is established. Finally, the satellite docking process requires pin–hole contact. These contact forms are crucial to the successful execution of the space assembly task, and the ability of the robot manipulator to transition seamlessly between these different forms is of utmost importance.

The adoption of the admittance control strategy in this paper is based on the inner loop position and allows for seamless switching between precise positioning and compliance control. The controller’s virtual spring effectively adjusts the error offset back to the desired trajectory. Therefore, this paper adopts the following admittance control strategy:

$$F_{e}=K\left(X_{r}-X\right)+B\left({\dot{X}}_{r}-\dot{X}\right)+M\left({\ddot{X}}_{r}-\ddot{X}\right)$$ (3)

where *X* is the actual pose of the end-effector and *X_r_* is the reference pose. *M* is the ideal inertia, *B* is the ideal damping of the robot, and *K* is the ideal stiffness of the robot. The impedance control adjusts *X* relative to the reference position and pose *X_r_* and according to the force *F_e_* measured by the sensor. In this way, we can achieve flexible operation of the manipulator.

When the manipulator comes into contact with the environment, the resulting interaction can be modeled as a typical second-order system [30], with a damping ratio of *ξ*. The admittance controller’s inertia and stiffness are denoted by *M_d_* and *K_d_*, respectively. Accordingly, the admittance controller damping can be expressed as:

$$B=\xi \cdot 2\sqrt{M_{d}K_{d}}$$ (4)

**Fig. 8.**
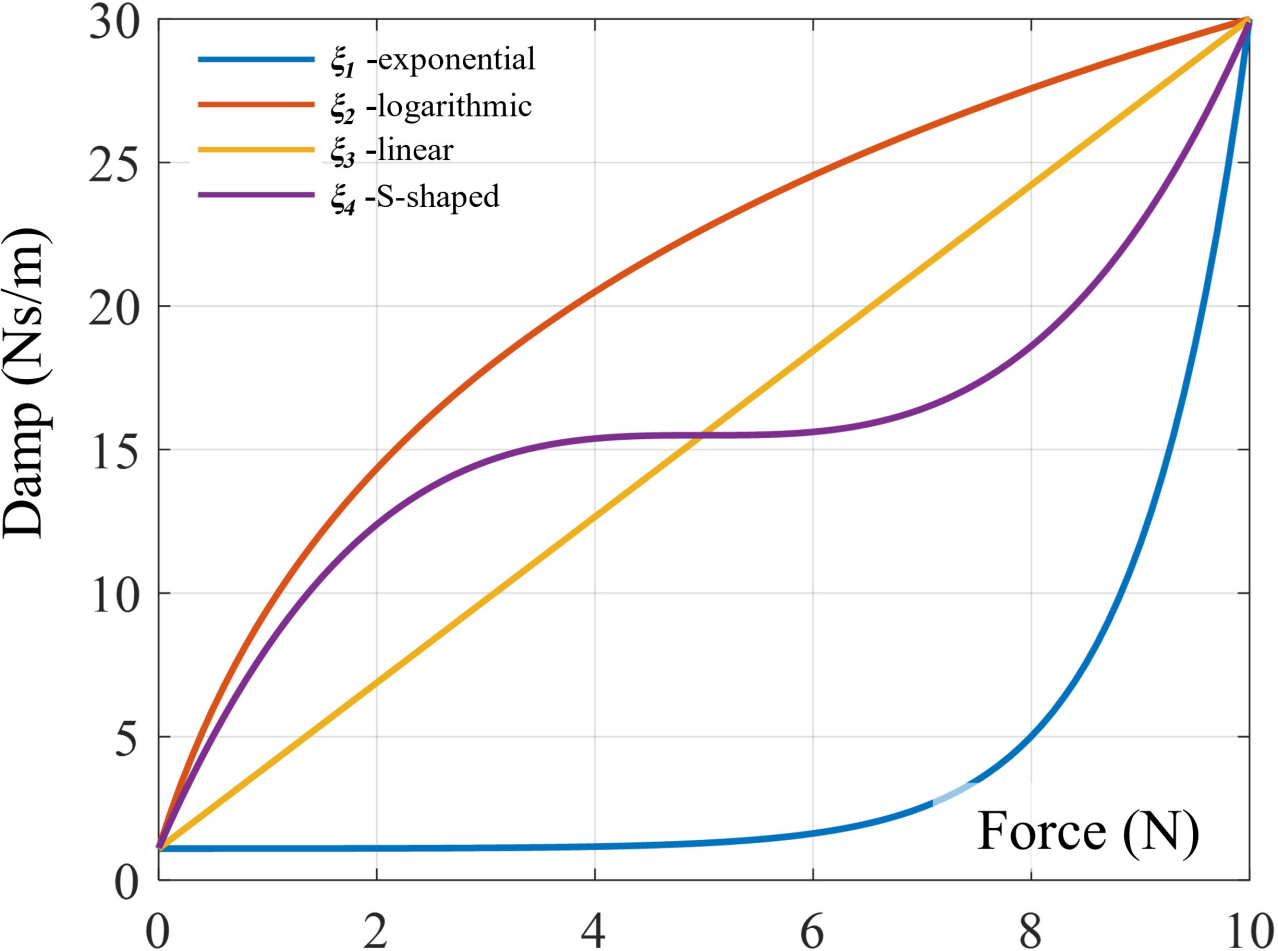
Curves of 4 damping variations.

To accurately reflect the system’s characteristics, we chose 4 mathematical expressions [i.e., exponential (Eq. 5), logarithmic (Eq. 6), linear (Eq. 7), and S-shaped (Eq. 8) curve expressions] to simulate different forms of variable damping compliance control. With the linear curve, the damping ratio increases linearly with the damping value and thus can provide a simple and predictable relationship between damping and contact force. The exponential curve has a slow increase in damping ratio for small damping values, but this increase becomes more rapid as the damping value increases. In the logarithmic curve, the damping ratio first increases rapidly for small damping values, but this increase slows down as the damping value increases (see Fig. 8).

The damping ratio (*ξ*) is a more intuitive parameter than the damping value, and establishing a relationship between *ξ* and contact force (*F*) is crucial for effective control of the system’s response. Therefore, by introducing these 4 mathematical expressions into the active compliance model, we can simulate various forms of variable damping compliance control and evaluate their effectiveness in improving system response.

$$\xi_{1}=k_{1}\left(e^{F}-1\right)+\xi_{0}$$ (5)

$$\xi_{2}=k_{2}\ln\left(F+1\right)+\xi_{0}$$ (6)

$$\xi_{3}=k_{3}F+\xi_{0}$$ (7)

$$\xi_{4}=k_{4}{\left(F-\frac{F_{m}}{2}\right)}^{3}+\frac{\xi_{m}+\xi_{0}}{2}$$ (8)

where *ξ*_1–4_ is the damping in different curves, *k*_1–4_ is the fixed coefficient, *F* is the contact force, *ξ*_0_ is the minimum damping, *ξ_m_* is the maximum damping, and *F_m_* is the maximum force. Given that all 4 curves pass through (0, *ξ*_0_) and (*F_m_*, *ξ_m_*), the fixed coefficient *k*_1–4_ is given by:

$$k_{1}=\frac{\xi_{m}-\xi_{0}}{e^{F_{m}}-1}k_{2}=\frac{\xi_{m}-\xi_{0}}{\ln\left(F_{m}+1\right)}k_{3}=\frac{\xi_{m}-\xi_{0}}{F_{m}}k_{4}=\frac{4\left(\xi_{m}-\xi_{0}\right)}{{F_{m}}^{3}}$$ (9)

**Fig. 9.**
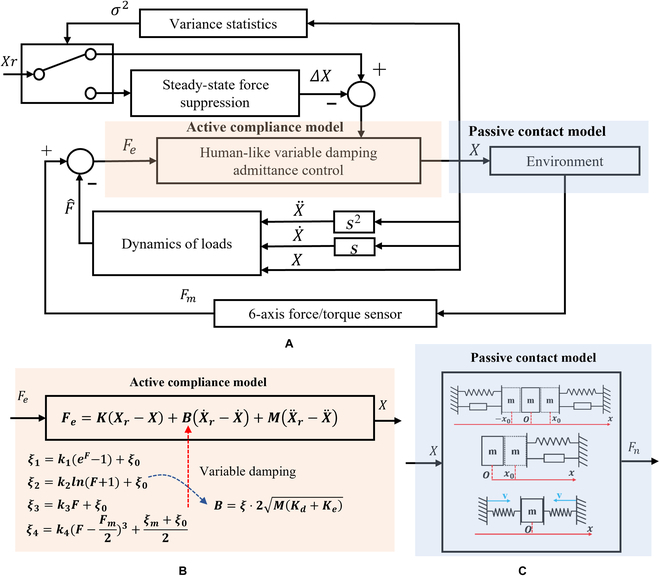
(A) Admittance control framework, (B) active compliance model, and (C) passive contact model.

The admittance control framework (Fig. 9A) is utilized to incorporate the damping ratio change law (Eqs. 5 to 8) into the damping term of the controller, which is used to map the active compliance model (Fig. 9B). In addition, 3 passive contact models from Table 1 are used to simulate the actual contact model (Fig. 9C). To tackle the issue of closed chain internal force in the steady state of compliance control, we propose a dynamic programming strategy based on the generalized forgetting factor function to enhance system stability.

## Simulation Experiments

### Simulation of human-like admittance controller

On the basis of the variations in damping presented in Fig. 8, we conducted simulations using 3 different contact models, as specified in Table 1. With identical initial conditions, we analyzed the impact of 6 groups of distinct controller parameters. The first 4 groups applied the aforementioned damping rules to the controller, while the final 2 groups had fixed damping ratios. For one of these last groups, the fixed damping ratio was the maximum damping ratio amplitude of the controller; for the other, the fixed damping ratio was set to the minimum damping ratio amplitude of the controller. The parameters used in the simulation included *k_d_* = 2, *m* = 0.2, and *k_e_* = 200 (*k_e_* denotes environmental stiffness); the damping ratio range was 1.1 to 30, with a lower limit of *ξ*_0_ = 1.1 and an upper limit of *ξ_m_* = 30. Because of the inherent delay between the measurement of external forces and the resulting actions of the actuator, we introduced a delay of 5 control cycles in the simulation.

**Fig. 10.**
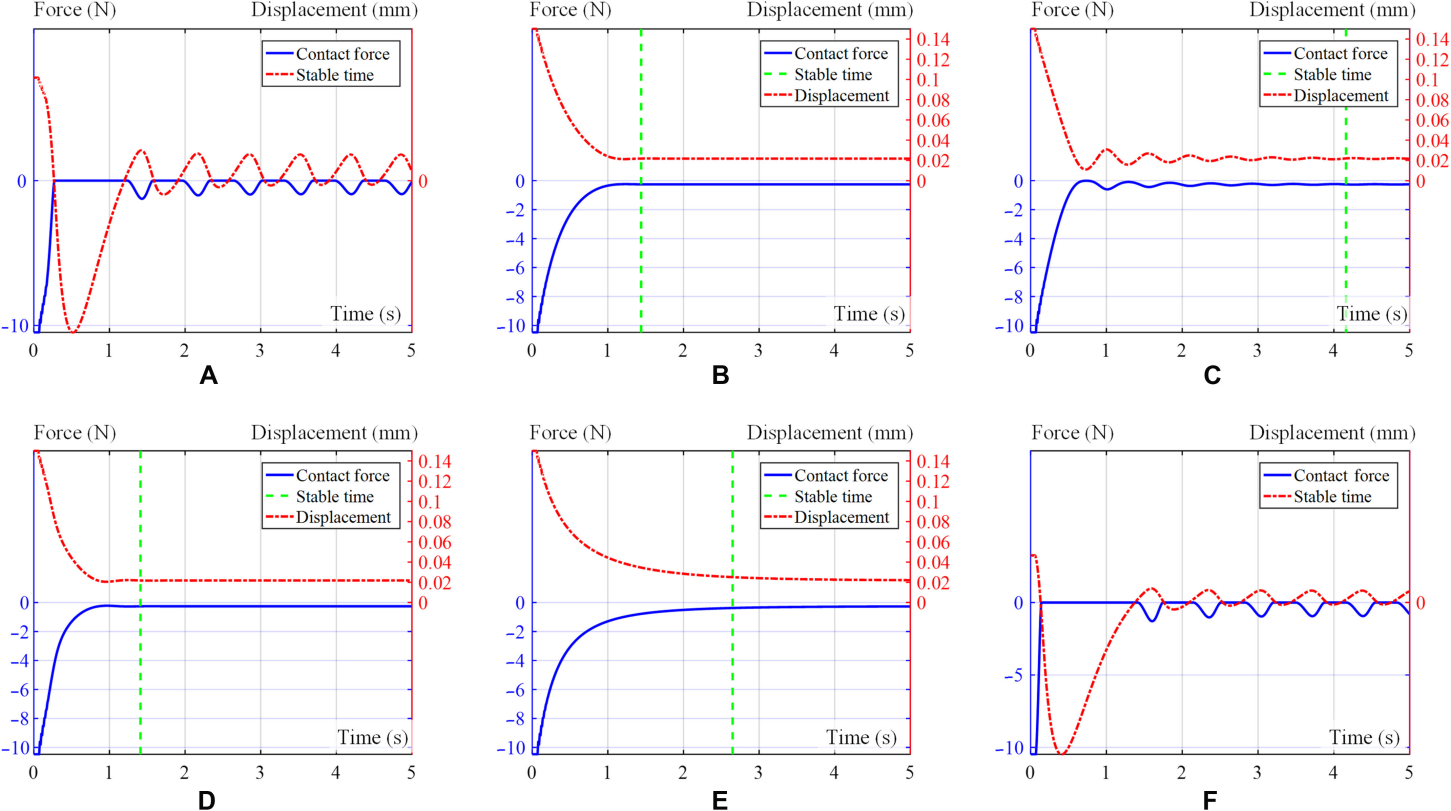
Curve of contact force and displacement in one-sided bevel contact form simulation. Exponential curve (A), logarithmic curve (B), linear curve (C), S-shaped curve (D), fixed damping *ξ_m_* (E), and fixed damping *ξ*_0_ (F).

**Fig. 11.**
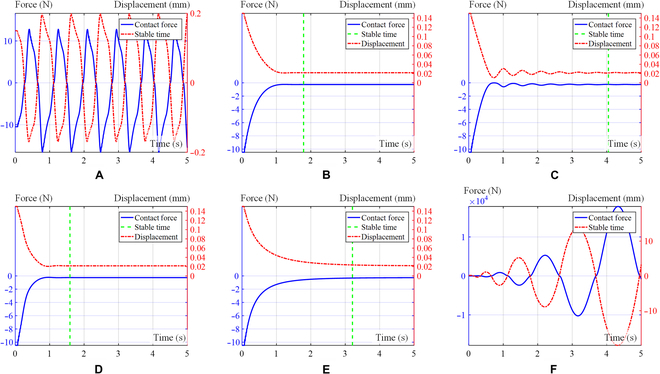
Curve of contact force and displacement in both sides bevel contact form simulation. Exponential curve (A), logarithmic curve (B), linear curve (C), S-shaped curve (D), fixed damping *ξ_m_* (E), and fixed damping *ξ*_0_ (F).

**Fig. 12.**
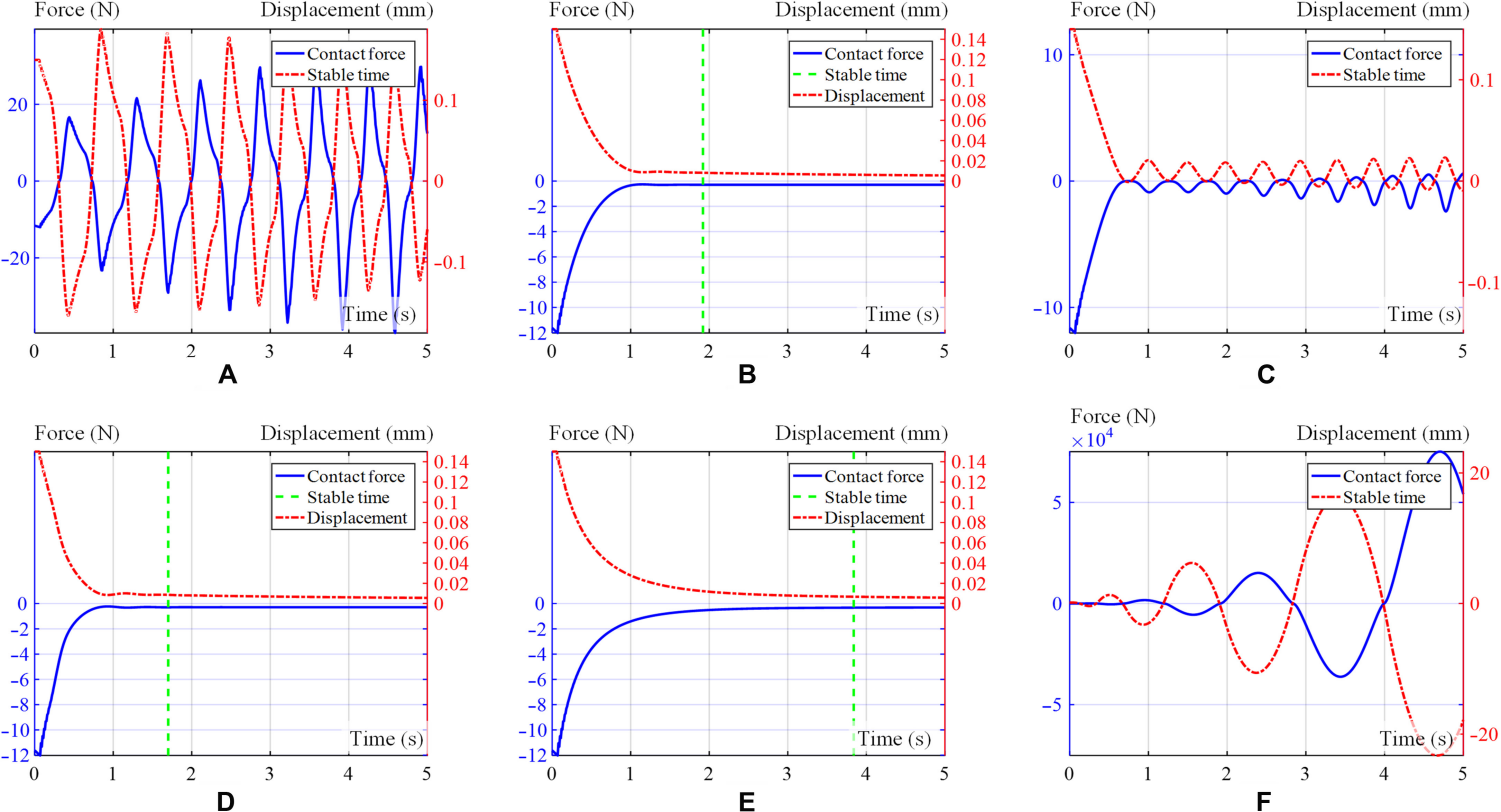
Curve of contact force and displacement in pin–hole contact form simulation. Exponential curve (A), logarithmic curve (B), linear curve (C), S-shaped curve (D), fixed damping *ξ_m_* (E), and fixed damping *ξ*_0_ (F).

1. One-sided bevel contact form. For the case of unilateral contact, as depicted in Table 1, assuming an initial position of *x*_0_ = 0.01, the expected initial position is *x* = 0.15. Figure 10 shows a visual representation of the contact force–displacement relationship observed in the 6 distinct parameter group. Figures 10 to 12 are the above corresponding relations: Exponential curve (A), logarithmic curve (B), linear curve (C), S-shaped curve (D), fixed damping *ξ_m_* (E), and fixed damping *ξ*_0_ (F), respectively. Figure 10 shows the effectiveness of the 4 variable damping controllers in reducing the velocity and in improving the dynamic response performance of a mass–spring–damper system under external excitation. These controllers are designed on the basis of different control strategies and can adjust the damping coefficient of the damper. To evaluate the time required to reach steady state, we use a time sequence to record the end displacement at each point in time. The steady state is considered to be achieved when the variance falls below a certain threshold and is given by:

$$\sigma^{2}=\frac{\sum_{i=1}^{n}{\left({\tilde{x}}_{i}-\bar{x}\right)}^{2}}{n-1}$$ (10)

where *x* = [*x*_1_, *x*_2_, …, *x_n_*], $\tilde{x}=\frac{\sum_{i=1}^{n}x_{i}}{\left|x\right|}=\left[{\tilde{x}}_{1},{\tilde{x}}_{2},\ldots ,{\tilde{x}}_{n}\right]$, and $\bar{x}=\frac{\sum_{i=1}^{n}x_{i}}{n}$.

We take *σ*^2^ < 10^−6^ as the steady-state criterion, and the time to reach this criterion is shown in Table 2.

2. Both sides bevel contact form. For the case of the both sides bevel contact form, as depicted in Table 1, we assume an initial position of *x*_0_ = 0.01, and the expected initial position is *x* = 0.15. Figure 11 shows the curve of displacement and contact force over time for the 6 distinct simulation groups. The environmental stiffness experiences a gradual increase from its initial value of *k_e_* = 200 to a maximum of 1,000 over a period of time. Figure 11 shows a visual representation of the contact force–displacement relationship observed in the 6 distinct simulation groups.
3. Pin–hole contact form. For the case of pin–hole contact, as depicted in Table 1, assuming an initial position of *x*_0_ = 0.01, the expected initial position is *x* = 0.15. Figure 12 provides a visual representation of the contact force–displacement relationship observed in 6 distinct simulation groups.

**Table 2.** Curves of stability time under various damping ratio changes (in seconds).

Contact form	Exponential	Logarithmic	Linear	S-shaped	Damping *ξ_m_*	Damping *ξ*_0_
One side	Unstable	1.47	2.15	1.25	2.69	Unstable
Both sides	Unstable	1.54	2.59	1.31	3.49	Unstable
Clamping	Unstable	1.50	Unstable	1.28	3.91	Unstable

After analyzing the figures and tables presented above, the contact force of the robot satellite assembly can reach a stable state under the damping S-shaped curve and has the fastest stable time compared to other damping change curves. It can be observed that the human-like admittance controller exhibits significant advantages in terms of rapid stability and effective suppression of system oscillations.

### Dynamic programming based on forgetting factor

**Fig. 13.**
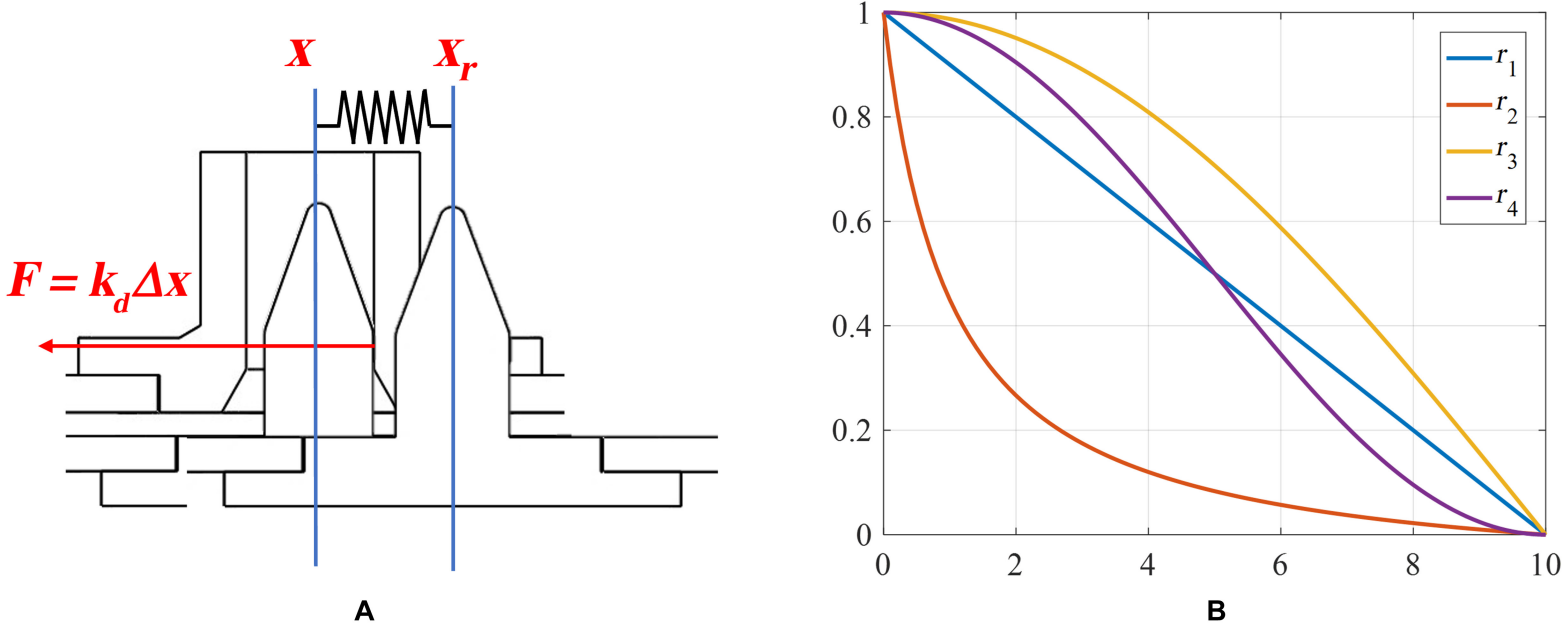
(A) Contact force generated by virtual spring and (B) 4 forgetting factor function curves.

The use of a human-like admittance controller can improve a robot’s ability to adapt to various environmental changes during the contact process, such as changes in contact force, by adjusting its stiffness and damping properties. This can enhance the stability of the contact process and potentially reduce the risk of damage to assembly components. However, as a result of potential errors in trajectory planning, the virtual spring’s equilibrium position may not align with the ideal docking location, leading to a substantial steady-state contact force (as illustrated in Fig. 13A). This excessive contact force causes a significant increase in the manipulator motor’s energy consumption; meanwhile, the large potential energy of the system makes it prone to vibration when the closed chain is released.

We introduce the forgetting factor function to the generalized control theory. The forgetting factor function is used to dynamically adjust the weights between the actual position and the steady-state expected position. The iterative formula is constructed as follows:

$$x\left(t\right)=\left(1-r\left(t\right)\right)x\left(t\right)+r\left(t\right)x_{r}$$ (11)

*r*(*t*) drops from 1 to 0 within time *T*. When *r*(*t*) = 0, the initial time is *t*_0_, and *x_r_* = *x*(*t*_0_ + *T*). Four forgetting factor functions are shown in Eq. 12, and their corresponding curves are shown in Fig. 13B (*t*_0_ = 0, *T* = 10). To simulate and observe the steady-state effect, we add these forgetting factor functions to the variable damping admittance controller with the aforementioned S-shaped change rules.

$$\begin{array}{cc} r_{1}\left(t\right) & =-\frac{t}{T}+1r_{2}\left(t\right)=\frac{T+1}{T\left(t+1\right)}-\frac{1}{T}r_{3}\left(t\right)=\cos\left(\frac{\pi }{2T}t\right)r_{4}\left(t\right)\\ & =\frac{1}{2}\cos\left(\frac{\pi }{T}t\right)+\frac{1}{2} \end{array}$$ (12)

**Fig. 14.**
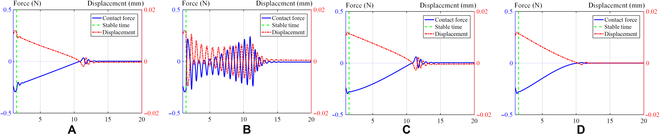
Comparison of 4 forgetting factor functions: (A to D) curves under the influence of weight functions *r*_1_ to *r*_4_, respectively.

To mitigate the instability of the contact process and avoid unnecessary interference with the contact force suppression control during steady state, we activate the forgetting factor function when the variance of the historical sequence dips below a specific threshold. Figure 14 shows the contact force and displacement curve with respect to time.

Upon analyzing the results, it is evident that the system’s stability is optimal when subjected to forgetting factor function *r*_4_. Table 3 shows the average values of the contact force historical sequence during the final time period for the 4 functions. The findings reveal that the controller using the forgetting factor function yields significantly lower contact force compared to the controller without it. Furthermore, the average contact force while utilizing the *r*_4_ function is the lowest.

**Table 3.** Final average contact force.

Curve type	None	*r*_1_	*r*_2_	*r*_3_	*r*_4_
Average contact force (N)	−0.2902	2.68 × 10^−3^	6.09 × 10^−3^	3.99 × 10^−3^	1.05 × 10^−4^

## Experiment Using a Ground Verification Platform

Robotic satellite assembly in ground and microgravity space environments involves many similarities and differences. In both environments, robots require precision and reliability in their operations, which places certain demands on the hardware platform’s accuracy. However, it is important to consider the differences between these 2 environments. In the ground environment, robots can rely on the stability and support provided by gravity to ensure precision and accuracy during the assembly process. In addition, because of the effect of gravity, robots are better able to control and position satellite components when manipulating them. In microgravity space environments, the lack of gravity means that robots must consider the inertial forces of the satellite itself during the assembly process. In this scenario, the satellite behaves like a floating mass system, presenting certain challenges for robot operations.

**Fig. 15.**
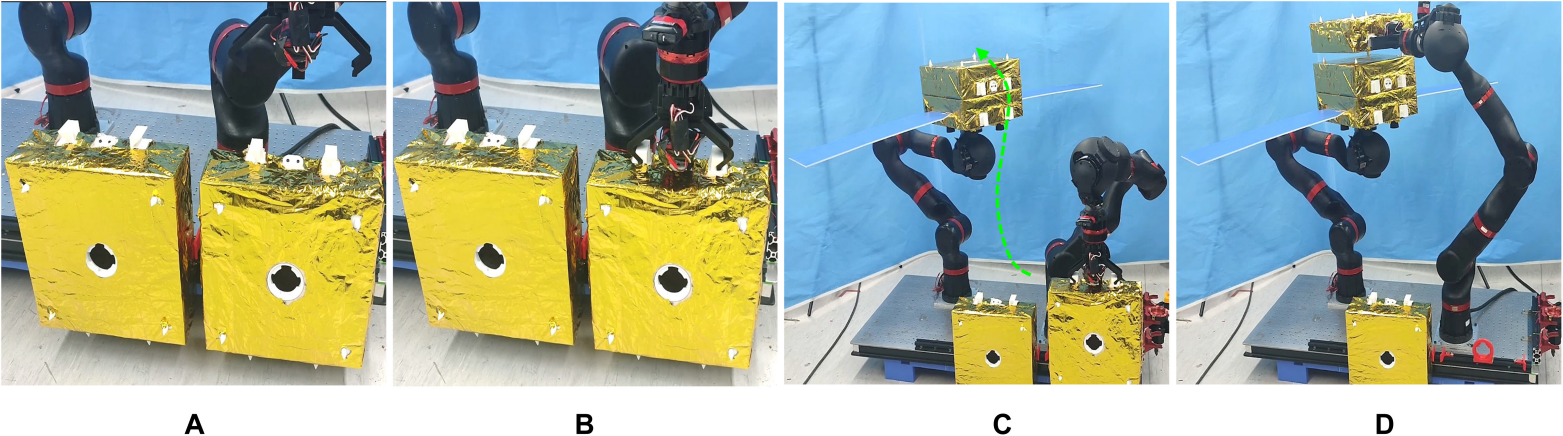
On-orbit maintenance experiment verification system of satellite. (A and B) Satellite capture process. (C and D) Satellite docking process.

The hand and arm of a humanoid robot are crucial components that have been extensively researched [31–32]. In this paper, we present a novel assembly method for a simulated robot satellite and develop a corresponding ground verification system scheme (see Fig. 15) that uses 2 manipulators called BIT-DMR [33]. One of the manipulators is specifically designed to replicate the microgravity conditions that a satellite would encounter in space, thereby counteracting the effects of gravity and enabling a weightless environment for the satellite to be assembled in. The second manipulator imitates the operational capability of a robotic astronaut and is responsible for executing the actual assembly process of satellite modules. These modules are secured in place by clamping jaws and pin–hole connections; meanwhile, the robot meticulously dismantles the prior module and precisely installs the new module.

Table 4 presents the stiffness and mass parameters of the admittance controller. To satisfy the prescribed lower and upper bound damping ratios of 1.1 and 30, respectively, we express the variation in damping for the 3 axes in the translational direction by Eq. 13. Likewise, Eq. 14 describes the change in damping for the 3 axes in the rotational direction.

$$\xi =0.115{\left(F-5\right)}^{3}+15.55$$ (13)

$$\xi =7.4{\left(\tau -1.25\right)}^{3}+15.55$$ (14)

**Table 4.** Admittance controller parameters.

Direction	*x*	*y*	*z*	*α*	*β*	*γ*
Stiffness K	4.00	4.00	4.00	1.00	1.00	1.00
Quality M	50.00	50.00	50.00	5.00	5.00	5.00

### Satellite capture tasks in satellite assembly

**Fig. 16.**
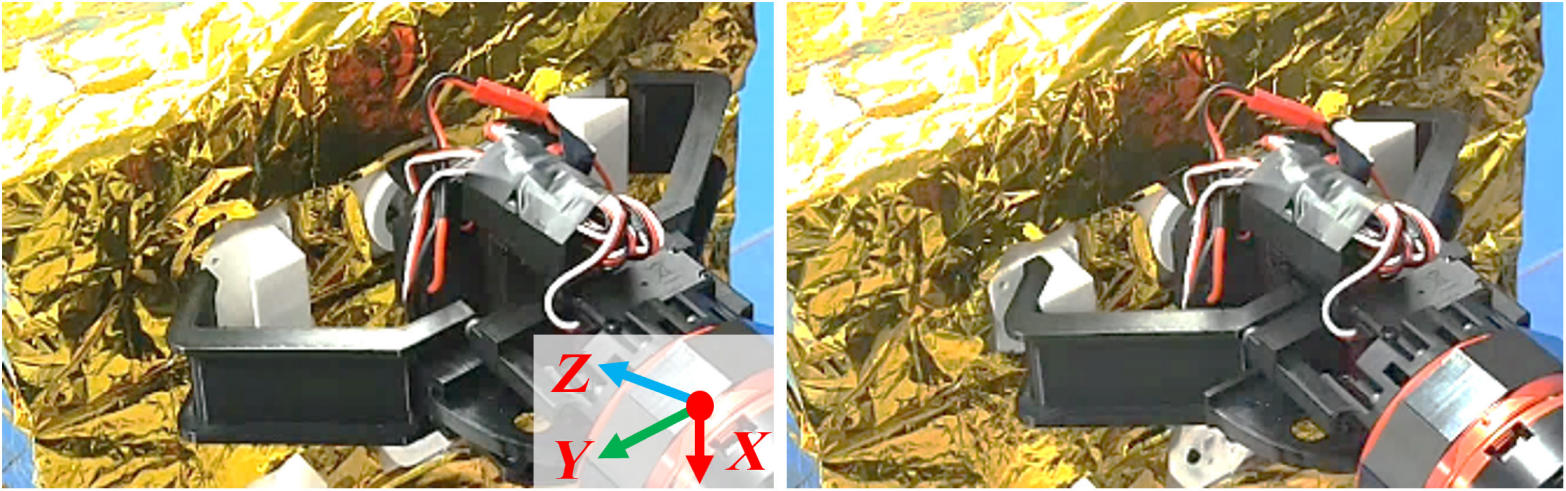
Satellite capture process.

As depicted in Fig. 16, the admittance control strategy enables the system to rectify any positioning deviations of the manipulator and successfully grasp the satellite. This is accomplished by leveraging the contact force observed during the gripper grasping process.

**Fig. 17.**
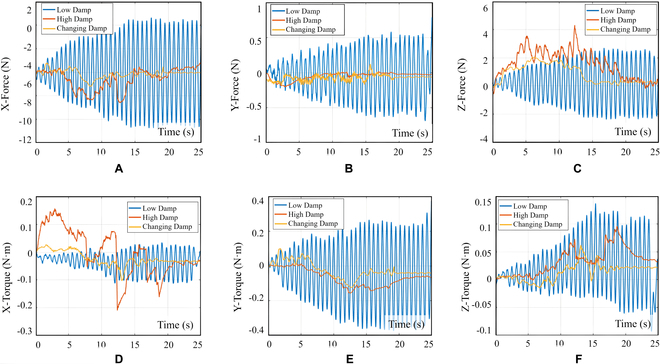
Curves of change with respect to different damping admittance controllers (gripper clamping). (A) Curves of contact force in the *X* direction. (B) Curves of contact force in the *Y* direction. (C) Curves of contact force in the *Z* direction. (D) Curves of contact torque in the *X* direction. (E) Curves of contact torque in the *Y* direction. (F) Curves of contact torque in the *Z* direction. The blue curve corresponds to the fixed damping admittance controller with a value of 1.1, the red curve corresponds to the fixed damping admittance controller with a value of 30, and the yellow curve represents the influence of the variable damping admittance controller with a value of *ξ*, as described in Eqs. 16 and 17.

Figure 17 illustrates the characteristics of the contact force and torque in 6 directions when the gripper is subjected to varying damping admittance control during the contact grasping point for the satellite capture process. The plotted curve shows that a small fixed damping leads to severe changes and oscillations in the contact force. Conversely, a large fixed damping results in slow response times for the manipulator, making it challenging to adapt to the increasing internal force of the closed chain in a timely manner. The proposed admittance controller, which is designed to mimic human-like movement, demonstrates significant efficacy in reducing contact force and in dampening vibrations during compliance control.

**Fig. 18.**
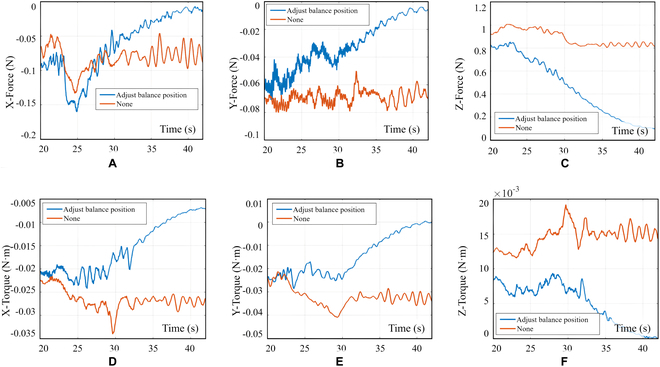
Change curves of the contact force (gripper clamping). (A) Curves of contact force in the *X* direction. (B) Curves of contact force in the *Y* direction. (C) Curves of contact force in the *Z* direction. (D) Curves of contact torque in the *X* direction. (E) Curves of contact torque in the *Y* direction. (F) Curves of contact torque in the *Z* direction. The red curve represents the contact force curve without dynamically adjusting the equilibrium position, while the blue curve represents the contact force curve with dynamic adjustment of the equilibrium position.

In this study, we compared the performance of 2 different control strategies for an admittance controller. Both strategies use the same damping variation pattern. The results showed that the dynamic equilibrium position adjustment strategy based on a forgetting factor was effective in reducing the steady contact force after the grasping process reached the steady state (see Fig. 18). This approach thus has the potential to improve the performance of admittance controllers for robotic grasping tasks.

### Satellite docking process in satellite assembly

**Fig. 19.**
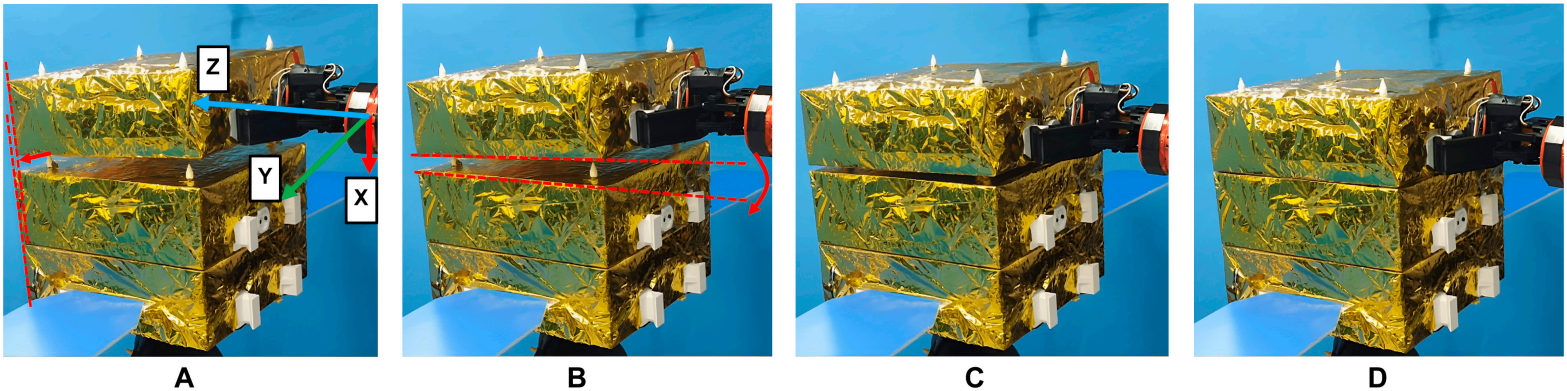
Module docking process. (A) Satellite module docking with initial position error. (B) Satellite module docking with angle initial error. (C) Satellite docking through compliant control strategy. (D) Complete module docking.

As shown in Fig. 19, the satellite docking process can automatically correct the initial pose error under the effect of compliance control strategy and hole guidance.

**Fig. 20.**
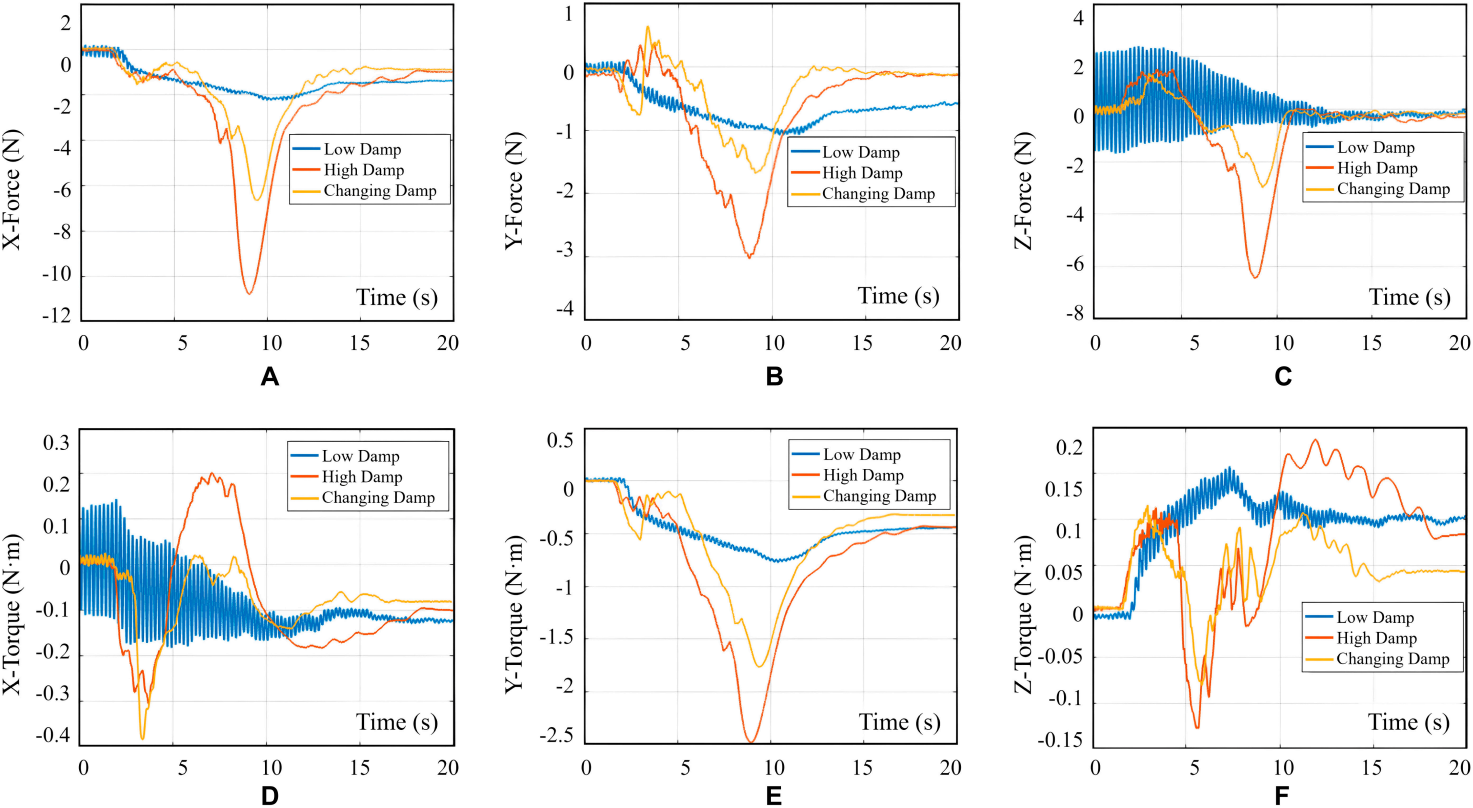
Contact force curves under different damping admittance controllers (satellite docking). (A) Curves of contact force in the *X* direction. (B) Curves of contact force in the *Y* direction. (C) Curves of contact force in the *Z* direction. (D) Curves of contact torque in the *X* direction. (E) Curves of contact torque in the Y direction. (F) Curves of contact torque in the *Z* direction. The blue curve corresponds to the fixed damping admittance controller with a value of 1.1, the red curve corresponds to the fixed damping admittance controller with a value of 30, and the yellow curve represents the influence of the variable damping admittance controller with a value of *ξ*, as described in Eqs. 16 and 17.

For the docking of a new satellite, we utilized the same controller parameters as those used in the gripper clamping process. Once the satellite was positioned at the docking preparation location, the manipulator was directed to move toward the docking position at a controlled speed of 5 mm/s. The contact force was recorded both before and after docking. As observed from the curve in Fig. 20, the variable damping admittance controller successfully suppressed the oscillations that occurred during the docking process. Furthermore, this controller could reduce the contact force while ensuring rapid response adjustment, thereby enhancing the safety of the docking process.

**Fig. 21.**
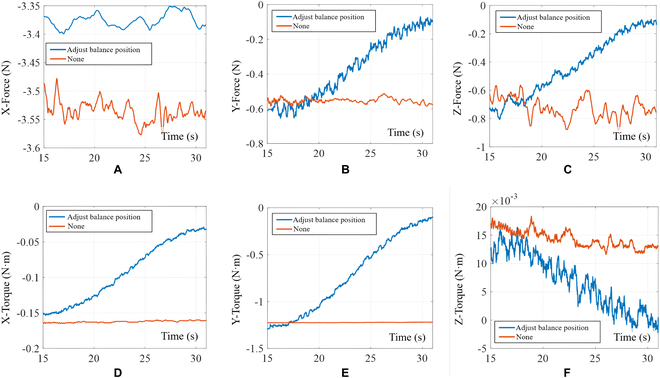
Contact force change curves (satellite docking). (A) Curves of contact force in the X direction. (B) Curves of contact force in the *Y* direction. (C) Curves of contact force in the Z direction. (D) Curves of contact torque in the X direction. (E) Curves of contact torque in the Y direction. (F) Curves of contact torque in the Z direction. The red curve represents the contact force curve without dynamically adjusting the equilibrium position, while the blue curve represents the contact force curve with dynamic adjustment of the equilibrium position.

To compare the effectiveness of the dynamic equilibrium position adjustment strategy based on the forgetting factor, we used an admittance controller with the same damping variation pattern (as shown in Fig. 21). The goal of the assembly was to maintain contact force in the *x* direction; therefore, the forgetting factor was not utilized to adjust the equilibrium position in that direction.

It is noteworthy that the task at hand and the gripper grasping task exhibit distinctive contact dynamics characteristics, notwithstanding the identical utilization of controller parameters. This underscores the remarkable adaptability of the human-like variable parameter admittance control strategy.

## Discussion

This paper aims to address 2 challenges in robot assembly: excessive internal force and inadequate adaptability. To overcome these issues, we establish active compliance and passive contact models in the assembly process by collecting human arm data. On the basis of these models, we develop a humanoid variable parameter admittance controller that exhibits excellent control performance in various assembly tasks. A ground verification platform was constructed to test the proposed control method in practical scenarios, including satellite grasping and assembly. The experimental results confirm the safety, robustness, and adaptability of the human-like simulated compliance variable parameter admittance control method.

In the context of space assembly operations, adopting humanoid strategies can bring several benefits. First, humanoid robots can provide greater flexibility and adaptability due to their ability to simulate human movements and actions. In space assembly operations, where robots need to perform complex and delicate tasks, such as docking modules and installing structural components, humanoid robots can better adapt to these tasks and successfully complete them. Second, humanoid robots can offer higher precision and accuracy by precisely simulating human hand movements and actions. This can ensure that the robot can accurately operate and control tools, such as screwdrivers and wrenches, to complete fine-assembly tasks. Third, adopting humanoid robots can improve the controllability and usability of the system. The human-like control interface can allow operators to use familiar operating methods and gestures to control the robot, thereby reducing operator training and learning costs.

Human-like control strategies can improve the adaptability, precision, and controllability of robots performing space assembly and maintenance tasks. These strategies are developed by collecting human arm data and establishing models that enable robots to simulate human-like compliance and adaptability. However, further research is necessary to enable robots to accomplish flexible assembly tasks comparable to real humans. Durable and reliable robots capable of withstanding harsh space environments are also needed. Advancements in humanoid control strategies can have important implications for the future of space exploration and development, thus improving mission efficiency, safety, and reliability.

## Acknowledgments

**Funding:** This work was supported by the National Natural Science Foundation of China (U22B2079, 62103054, U1913211, U2013602, and 62273049). **Competing interests:** The authors declare that they have no competing interests.

## Data Availability

The data are available from the corresponding author on reasonable request.
